# Nanocellular Polymers: The Challenge of Creating Cells in the Nanoscale

**DOI:** 10.3390/ma12050797

**Published:** 2019-03-07

**Authors:** Judith Martín-de León, Victoria Bernardo, Miguel Ángel Rodríguez-Pérez

**Affiliations:** CellMat Laboratory, University of Valladolid, Paseo de Belen 7, 47011 Valladolid, Spain; vbernardo@fmc.uva.es (V.B.); marrod@fmc.uva.es (M.Á.R.-P.)

**Keywords:** polymethylmethacrylate, sepiolites, poly(methyl methacrylate)-poly(butyl acrylate)-poly(methyl methacrylate), nanocellular polymer, homogeneous nucleation, heterogeneous nucleation, gas dissolution foaming

## Abstract

The evolution of technology means that increasingly better materials are needed. It is well known that as a result of their interesting properties, nanocellular polymers perform better than microcellular ones. For this reason, the investigation on nanocellular materials is nowadays a very topical issue. In this paper, the different approaches for the production of these materials in our laboratory are explained, and results obtained by using polymethylmethacrylate (PMMA) are shown. Homogeneous nucleation has been studied by using raw PMMA, while two different systems were used for heterogeneous nucleation; adding nanoparticles to the system and using nanostructured polymers as solid precursors for foaming. The effects of the different parameters of the production process (gas dissolution foaming process) have been evaluated for all systems being possible to establish a comparison between the materials produced by different approaches. Moreover, the limitations and future work to optimise the materials produced are also discussed.

## 1. Introduction

Cellular solids are two-phase materials in which a gas or a liquid is dispersed in a continuous solid phase. These materials appear widely in nature, and they are also manufactured on a large scale by man [1]. Cellular solids present an exciting combination of properties, such as being lightweight (which allows energy and cost savings), having high energy absorption, and having low thermal conductivity. If the solid phase is a polymer, the material is said to be a polymeric cellular material or a cellular polymer. 

Cellular polymers show a variety of properties, such as buoyancy, chemical resistance, skin friendliness, no water absorption, cushioning performance, shock absorption, thermal insulation, and being lightweight [2,3,4]. These properties have promoted its widespread use in technological sectors such as the automotive and aeronautical industries, renewable energies, construction, biotechnology, cushioning, and packaging. The cellular polymer market is a relevant economic sector, and it is estimated to reach $126.08 billion by 2022 at an annual growth rate of 5.86% [5].

Cellular polymers can be classified according to their average pore size. For instance, conventional cellular polymers show a wide range of cell sizes, from a few millimeters to tens of micrometers. The properties of such materials are mainly controlled by their relative density and the chemical composition of the polymeric matrix [1]. Nonetheless, when the cell size is reduced below 10 μm (microcellular polymers), a significant improvement in the mechanical properties of these materials is observed [6,7,8,9]. Since their development at the Massachusetts Institute of Technology (MIT) in the early 1980s, the use of microcellular polymers has expanded to cover a significant number of applications, as the density reduction does not imply an abrupt decrease in mechanical properties for these systems.

In this area of research, the next generation of cellular polymers are the so-called nanocellular polymers, characterized by cell sizes in the nanometric range [10,11]. These materials have aroused great interest in the scientific community due to their new properties and promising future applications. To begin with, different mechanical, acoustic, and dielectric properties have been found in nanocellular polymers, compared with microcellular materials [12,13,14]. These behaviours can be attributed to the confinement of the polymer in very thin cell walls, an effect that has been confirmed using different techniques such as differential scanning calorimetry (DSC) or Raman spectroscopy [15,16]. In addition, such materials are present very low thermal conductivities due to the so-called Knudsen effect [17,18,19,20]. According to the Knudsen theory [21], when the cell size is comparable to the mean free path of the gas molecules inside the pores (around 70 nm), the gas molecules collide more often with the cells walls than with each other, so the energy transfer through the gas is reduced. Therefore, the conduction throughout the gaseous phase is considerably reduced while decreasing the cell size. This effect results in materials with thermal conductivities much lower than conventional cellular polymers or microcellular materials, thus providing a new generation of materials with heat transfer reductions of a factor of 2–3, which will allow energy savings and the reduction of wall thickness in buildings.

Moreover, due to their nanometric cell size and the high specific area associated with these nanopores, these nanocellular materials could also be employed in some specific applications in which other cellular materials cannot be used, such as membranes for micro and ultrafiltration applications or in catalysis and sensors [22,23,24]. In addition, the nanostructured surfaces generated in these materials are also suitable candidates for surface nano-functionalization for biomedical applications [25,26,27]. Finally, it has been proven that if the initial solid material is an amorphous transparent polymer, the nanocellular material (with sizes below 50 nm) could keep, up to some extent, the transparent character of the former solid [28]. Therefore, these novel materials could be used to produce materials that do not exist nowadays—that is, semi-transparent materials with low thermal conductivities and a significant toughness [29].

In short, the potential field of application of nanocellular polymers is broad and promising. Furthermore, new, unexpected effects with surprising applications might appear due to the dimensions of the cell walls and cells within the nanometric range.

The aim of this paper is to provide a comprehensive analysis of our research on this topic, summarizing some key results obtained and comparing for the first time in the same work the two different approaches (homogeneous and heterogeneous nucleation) that can be followed to create these new materials. In addition, the future approaches that could be used to improve the developed materials are discussed.

## 2. Production of Nanocellular Polymers

Despite the exciting properties of nanocellular polymers, its production is still nowadays a challenging task for scientists around the world. On the one hand, the production of nanocellular polymers has been achieved for different polymer matrices such as polymethylmethacrylate (PMMA) [30], polycarbonate (PC) [31], thermoplastic polyurethane (TPU) [32], polyetherimide (PEI) [33], and polyphenylsulfone (PPSU) [34], but there exist many other systems, such as polystyrene (PS), in which the production of nanocells has not yet been reported. Moreover, the production of nanocellular polymers in the large scale is not yet a reality due to the difficult task of producing large and homogeneous parts of such materials. It has not been until recent years that technological development has allowed the manufacturing of nanocellular polymers with enough dimensions to test and verify their properties [10].

The primary challenge in the fabrication of nanocellular polymers is that it requires specific production routes for creating and growing pores in the nanoscale. The techniques employed are very diverse and can be divided into three large groups: Phase separation techniques, imprinting or templating approaches, and the foaming method [10,35]. In the phase separation techniques, a solvent is used to produce a phase-separation process and generate the pores [36,37]. This separation can be induced chemically, thermally, or by using an immersion technique. In the imprinting/templating methods, the polymer is arranged following a nanometric pattern, using a self-organized media as a template which is reproduced in order to obtain the porous polymer [36,37]. In the third approach, foaming processes, gases are used to generate the pores [11,16,31,38,39]. While other techniques are generally restricted to the fabrication of thin films and require the use of organic solvents that have to be removed after producing the cellular material, foaming allows producing larger samples without the use of solvents.

One of the most promising foaming techniques is the gas dissolution foaming [6]—carbon dioxide (CO_2_) gas dissolution foaming, in particular. This method consists of dissolving CO_2_ in a polymer to generate the cellular structure. This gas is an excellent choice because of its outstanding diffusion characteristics in the supercritical state and the relatively mild conditions to reach this state (31 °C and 7.3 MPa). Furthermore, carbon dioxide is a green solvent that can be removed without leaving any residue or the production of any pollutant compound. 

The gas dissolution foaming process consists of three stages. Firstly, a sample is introduced in a pressure vessel at specific conditions of pressure and temperature. CO_2_ diffuses into the sample until the maximum amount of gas is dissolved in the polymer (saturation). The amount of gas absorbed depends on both the pressure and the temperature of the gas [40,41]. During saturation, CO_2_ plasticizes the polymer, and its glass transition temperature drops [42,43,44,45]. The polymer is now characterized by its effective glass transition temperature, T_g,eff_. After saturation, the pressure is released, and the sample is supersaturated; that is, the amount of gas inside the sample is much higher than the equilibrium concentration at room temperature. Finally, foaming takes place. If the saturation temperature is higher than the T_g,eff_ (i.e., the polymer is in the rubbery state), the foaming process occurs during the pressure release. In this case, the process is usually called one-step gas dissolution foaming [46]. Otherwise, foaming is promoted by heating the sample in a thermal bath at a temperature above the T_g,eff_. This process is called two-step gas dissolution foaming [6].

The physics underlying this process are more complicated than they may look. To begin with, pores must be created. The appearance of pores from a gas/polymer mixture is called phase-separation; that is, the one-phase gas/polymer system evolves to a two-phase system. The number of pores required to obtain a nanocellular material is huge. Consider Equation (1), relating the cell density $N_{v}$ (number of pores per cubic centimetre), the porosity of the cellular material $V_{f}$, and the cell size ϕ [6,47].

(1) $$V_{f}=\frac{\pi \phi^{3}}{6}N_{v}$$

According to this equation, to obtain a nanocellular material with an average cell size of 200 nm and a porosity of 0.9, the cell density must be approximately 2 × 10^14^ cells/cm^3^. If we assume that there is no coalescence, we can calculate the number of initial nuclei in the solid material prior to expansion—that is, the cell nucleation density $N_{0}$ (Equation (2)). For this particular example, the cell nucleation density should be of the order of 2 × 10^15^ nuclei/cm^3^.

(2) $$N_{0}=\frac{N_{v}}{1-V_{f}}$$

Here arises one of the main challenges in the production process of nanocellular materials: A large number of cells must be created—higher than 10^14^ nuclei/cm^3^—and these pores must grow up to the required cell size and relative density without coalescence (i.e., keeping the very high number of cells). The creation and stabilization of these nanometric cells are the key physical mechanisms that must be understood and controlled to produce these novel materials.

### 2.1. Phase-Separation Mechanisms

During the formation of a new phase, such as the formation of a pore in a polymer, two major mechanisms can occur: Spinodal decomposition, nucleation, and growth, with the latter being usually considered as the primary mechanism in most of the foaming processes [48]. The gas/polymer system after saturation in the solid-state foaming process is in a supersaturated state, which is metastable. Phase-separation is induced by a sudden change in the thermodynamic conditions, such as a pressure release or an increase of temperature.

#### 2.1.1. Spinodal Decomposition

For specific concentrations of gas and ambient conditions, the gas/polymer system—that is, the polymer with the CO_2_ dissolved—undergoes spontaneous phase separation without the appearance of nucleation points [49]. This mechanism, called spinodal decomposition, takes place at high supersaturations at which the mixture is unstable [50]. This phenomenon is characterized by the vanishing of the energy barrier of nucleus formation [51], so gas molecules immediately start to form clusters which rapidly grow and coalesce. The result of the spinodal decomposition is a single gas phase or, in other words, an interconnected or co-continuous cellular structure [31,33,48].

#### 2.1.2. Homogeneous Nucleation

Nucleation and growth are believed to be the major mechanisms controlling the formation of a cellular structure in the gas dissolution foaming process. Nucleation is a phase-separation process which consists of the appearance of small clusters or aggregates of gas (nuclei) in a gas/polymer mixture after a sudden change in the thermodynamic conditions [50,51]. Then gas diffuses from the gas/polymer melt to the newly created nuclei. As more gas goes into a nucleus, it grows, and the final pore is obtained. As opposed to spinodal decomposition, nucleation implies an energy barrier that must be overcome in order to create a nucleus.

In a pure gas/polymer mixture (that is, without any other phase and/or impurities), nucleation is said to be homogeneous. According to Classical Nucleation Theory (CNT), the Gibbs free energy barrier ($\Delta G_{hom}$) that a nuclei should overcome to grow into a bubble depends on the surface tension between the pore and the polymer phase (γ) and the pressure difference between gas and solid (Δp), according to Equation (3) [51]:

(3) $$\Delta G_{hom}=\frac{16\pi \gamma^{3}}{3\Delta p^{2}}$$

From this equation, it is possible to obtain the critical value of the nuclei radius, $r_{c}$ (Equation (4)) [52,53,54]. Any cluster of gas molecules smaller than the critical radius will not be stable, whereas nuclei with sizes larger than the critical radius will survive and grow. The critical radius has been estimated several times for different systems [55,56,57], and it takes values between 0.2 and 8 nm.

(4) $$r_{c}=\frac{2\gamma }{\Delta p}$$

The homogeneous nucleation rate N is given by Equation (5), where $C_{0}$ is the initial concentration of gas in the polymer, $f_{0}$ is the frequency factor of gas molecules joining the nucleus, $k_{B}$ is the Boltzmann constant, and T is the temperature [52,58]:

(5) $$N=f_{0}C_{0}\;\exp\left(-\frac{\Delta G_{hom}}{k_{B}T}\right)$$

It has been established that this CNT model severally under-predicts the nucleation rate for homogeneous nucleation [53]. However, this theory provides a useful insight into the parameters involved in the nucleation process. 

To begin with, the amount of gas dissolved in the polymer affects nucleation, and the nucleation increases as the gas concentration increases. Thus, to obtain the high nucleation ratios needed to obtain a nanocellular material, high amounts of gas must be dissolved into the polymer. This relation between the concentration of gas and the cell density has been found experimentally in numerous studies with homopolymers [31,34,39,56,59]. It seems that there is a critical CO_2_ concentration for the formation of a nanocellular polymer [31,34,39,59]. The solubility of a gas into a polymer depends on both the pressure and the temperature.

On the one hand, solubility increases with pressure. However, the dependency of the solubility with pressure is different for each material. Three different situations are possible. Both quantities can be correlated linearly (Henry’s law), potentially (Langmuir’s model), or using a dual model which considers both contributions [40]. On the other hand, solubility decreases exponentially with temperature following an Arrhenius equation [41] for systems in which the sorption of gas is an exothermic process. In short, high pressures and/or low temperatures are required to obtain nanocellular polymers using the homogeneous nucleation mechanism.

Both temperature and pressure gradient play a role in the nucleation process according to Equations (3) and (5). Higher temperatures will lead to higher nucleation ratios [16], whereas a higher pressure gradient will also induce a larger nucleation rate [60]. Finally, the interfacial surface tension of the polymer also plays a role in the Gibbs free energy barrier, and this parameter is related with the viscosity of the polymer [61], which is also known to affect nucleation [48].

In short, a fine control of the processing parameters is mandatory for the optimum production of nanocellular materials based on homopolymers.

In order to understand all the key parameters for the production of a nanocellular homopolymer, we have studied each process parameter in depth by analysing how the modification of each one affects the cellular structure.

As was aforementioned, it has been found that there exists a critical amount of gas uptake that allows the cellular structure to evolve from microcellular to nanocellular. In our laboratory, two different systems have been studied to verify this fact. It has been proven that both polymethylmethacrylate (PMMA) and polyphenylsulfone (PPSU) present a critical solubility above which they present cells in the nanometric range. 

This critical solubility strongly depends on the polymeric matrix: While PPSU only requires between a 9 and 9.5 wt.% of CO_2_ to present nanocellular structures [34], in PMMA the solubility limit increases to around 31 wt.% [13]. 

To deepen the understanding of the effect of the gas absorbed, we have focused our efforts on PMMA. In order to achieve this high amount of gas dissolved, we have tested the two strategies previously mentioned: The use of high saturation pressures and low saturation temperatures. On the one hand, a pressure of 31 MPa was used with a saturation temperature of 25 °C. These saturation conditions led to a 31 wt.% of gas uptake in PMMA [16]. On the other hand, with a fixed saturation temperature of −32 °C, three saturation pressures were tested: 6, 10, and 20 MPa. The abrupt decrease of the saturation temperature resulted in a drastic increase of the solubility, obtaining 39.7, 40.9, and 47.7 wt.% of CO_2_ absorbed for the different pressures used, respectively [29]. The obtained solubility by using 20 MPa of saturation pressure and −32 °C of saturation temperature (47.7 wt.%) was the highest ever reported for PMMA (this result was obtained using commercial PMMA V825 T supplied by Altuglass®, Philadelphia, PA, USA). 

**Figure 1:**
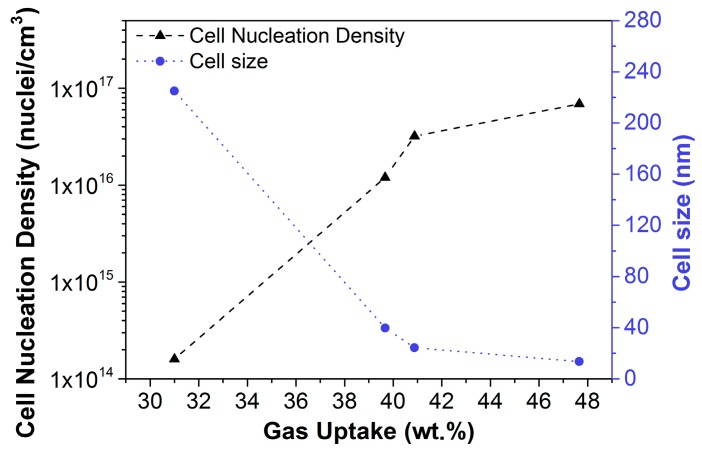
Cell nucleation density (left axis) and cell size (right axis) as a function of the solubility for homogeneous polymethylmethacrylate (PMMA).

With the aim of studying how this change in the solubility affects to cellular material, the cell size and the cell nucleation density were characterized. Materials with a similar relative density (around 0.4) were chosen for this study. The obtained results are shown in Figure 1.

As can be seen in Figure 1, both the cell size and the cell nucleation density suffered important changes with the increase of the solubility. The cell size was drastically reduced when passing from 31 to 40 wt.% from 225 nm to 40 nm. Moreover, when the solubility rose to a 48 wt.%, the cell size becomes even smaller (14 nm).

Otherwise, the cell nucleation density increased by two orders of magnitude when the solubility reached 40 wt.%. With the maximum amount of gas uptake, a cell nucleation density as high as 1 × 10^17^ nuclei/cm^3^ was reached.

In conclusion, by taking full advantage of high pressures and low saturation temperatures, we have been able to produce nanocellular PMMA with the lowest cell size and the highest maximum cell nucleation density ever reported.

**Figure 2:**
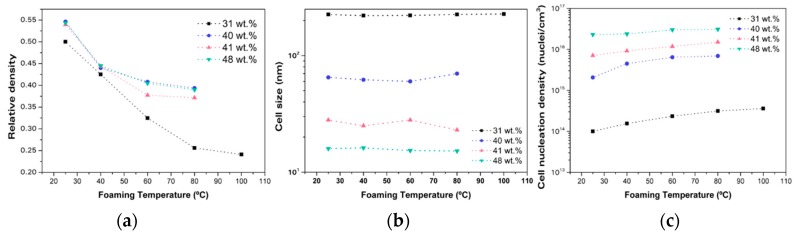
(**a**) Relative density as a function of the foaming temperature for 1 min of foaming time for homogeneous PMMA; (**b**) cell size as a function of the foaming temperature for 1 min of foaming time for homogeneous PMMA; and (**c**) cell nucleation density as a function of the foaming temperature for 1 min of foaming time for homogeneous PMMA.

The effects of the foaming parameters were also tested by doing experiments with different foaming temperatures and times. Figure 2a shows the change in the relative density as a function of the foaming temperature for the different solubilities. These results were obtained when we used 1 min of foaming time. The results were very similar for foaming times of 2 min and 5 min.

From this graph, it can be concluded that it is harder to reduce the density for samples with a higher solubility. While for lower foaming temperatures (25 °C and 40 °C), the relative density of the samples were similar despite the solubility, when the foaming temperature was increased, the relative density of the samples with higher solubility reached equilibrium; for samples containing a 31 wt.% of gas uptake, it continued decreasing. In fact, the relative density of samples with the highest solubility decreased from 0.55 to 0.45, but a further increase of the foaming temperature slightly decreased the relative density, reaching an equilibrium relative density of around 0.4. However, the behaviour of the samples with solubility of 31 wt.% was different. An increase in the foaming temperature from 25 °C to 80 °C resulted in an important reduction of the relative density (from 0.5 to 0.25)—this temperature allows these samples to reach their equilibrium density. 

This change in relative density is caused by a modification in the cellular structure, as can be seen in Figure 2c. While the increase of the foaming temperature does not have any effect in the cell size (Figure 2b), the cell nucleation density suffers important changes. It can be observed that the tendency of the cell nucleation density with foaming temperature is the opposite to that of the relative density. Therefore, it can be concluded that an increase of the cell nucleation density due to the higher foaming temperature is the primary mechanism responsible for the decrease in relative density. 

#### 2.1.3. Heterogeneous Nucleation

When some interphases are already present in the gas/polymer mixture, the nucleation process tends to take place in these pre-existing surfaces. This process is called heterogeneous nucleation. As in the case of homogeneous nucleation, the heterogeneous nucleation rate is mainly determined by an energy barrier $\Delta G_{het}$, which can be written as follows according to CNT [58]:

(6) $$\Delta G_{het}=\frac{16\pi \gamma^{3}}{3\Delta p^{2}}f\left(\theta \right)$$

where f(θ) is the ratio between homogeneous and heterogeneous nucleation. This function is always less than or equal to one [51]; that is, the Gibbs free energy barrier for heterogeneous nucleation is lowered, so nucleation is enhanced compared to the homogeneous situation. The function f(θ) can be written in terms of the wetting angle θ of the polymer-additive-gas interface [58]:

(7) $$f\left(\theta \right)=\frac{1}{4}\left(2+\cos\theta \right){\left(1-\cos\theta \right)}^{2}$$

**Figure 3:**
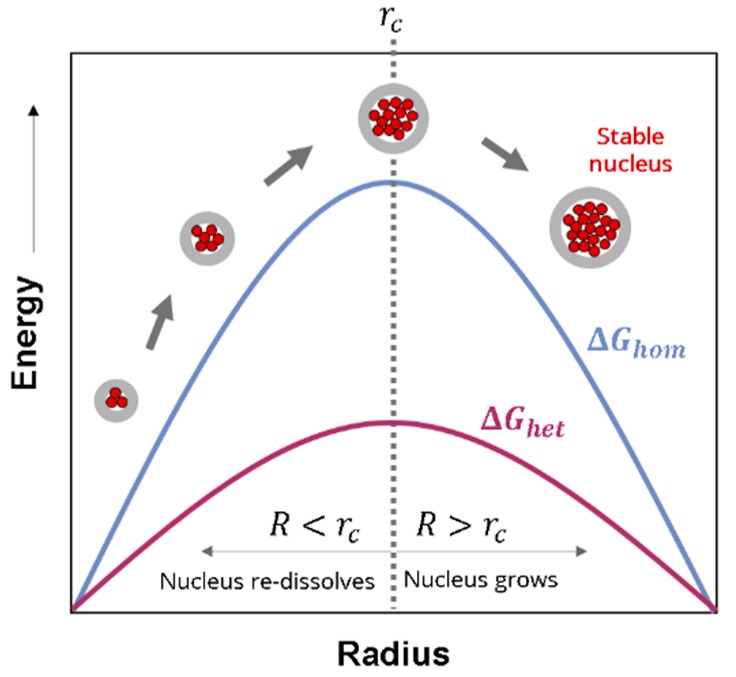
Schematic representation of the energy as a function of the nucleus radius, adapted from reference [63].

Thus, the addition of a second phase provides heterogeneous surfaces on which the nucleation energy barrier is lowered. For instance, the addition of particles can reduce the energy barrier in a factor of a thousand [62]. Two types of additives can be used as nucleating species: Inorganic particles or organic phases such as nanostructured polymers. Figure 3 shows a graphical scheme of the nucleation process, presenting the Gibbs energy as a function of the nucleus radius for both the homogeneous and the heterogeneous nucleation. If the radius is higher than the critical radius $r_{c}$, then the nucleus grows and becomes stable. Otherwise, nuclei smaller than $r_{c}$ re-dissolve in the mixture and do not result in a cell. Regarding the differences between homogeneous and heterogeneous nucleation, Figure 3 shows that the barrier for nucleus formation is smaller with the presence of heterogeneous species.

The heterogeneous nucleation rate $N_{het}$ is given by Equation (8), where $C_{1}$ is the initial concentration of gas in the polymer and $f_{1}$ is the frequency factor of gas molecules joining the nucleus.

(8) $$N_{het}=f_{1}C_{1}\;\exp\left(-\frac{\Delta G_{het}}{k_{B}T}\right)$$

The addition of particles promotes the formation of a nanocellular material as long as the density of the particles in the solid material is of the same order of magnitude as the nucleation density required, and the solid particles are active as nucleation agents. For this purpose, nanoparticles are needed, as their reduced size produces a higher density of nucleation points at the same particle concentration than micron-sized particles. In order to promote the growth of stable nuclei, particle size should be at least of the same size as the critical nucleus diameter but smaller than the desired cell size [55]. 

The addition of nanoparticles to enhance the nucleation density has been investigated in the development of micro and nanocellular materials. A wide variety of polymeric matrices and nanoparticles have been used for this purpose [62,64,65,66,67,68,69]. In particular, our group has recently discovered a new promising system able to produce nanocellular polymers: PMMA-sepiolites nanocomposites [70].

Sepiolites are needle-like nanoparticles with an average particle length ranging between 1 and 2 µm and a diameter in the nanometric range (between 20 and 30 nm) [71,72]. These clays chemically correspond to hydrated magnesium silicates (formula Si_12_Mg_8_O_30_(OH)_4_(OH_2_)_4_·8H_2_O). These clays have been shown to be appropriate inorganic nanofillers for polymers because of their diameter dimensions at the nanoscale and their high aspect ratio [73,74,75,76,77,78,79,80]. Their nanometric diameters make them suitable candidates for the production of nanocellular polymers. In our work, these nanometric sepiolites have been used for the first time as nucleating agents for the production of PMMA-based nanocellular polymers [70]. Solid nanocomposites based on PMMA with sepiolites have been produced by extrusion. Three different sepiolites were employed: A non-organically modified sepiolite (S-N) and two superficially organomodified sepiolites, one modified with a quaternary ammonium salt (S-QAS) and another modified with a silane (S-S). The process to obtain and modify these particles can be found elsewhere [74,80,81]. 

**Figure 4:**
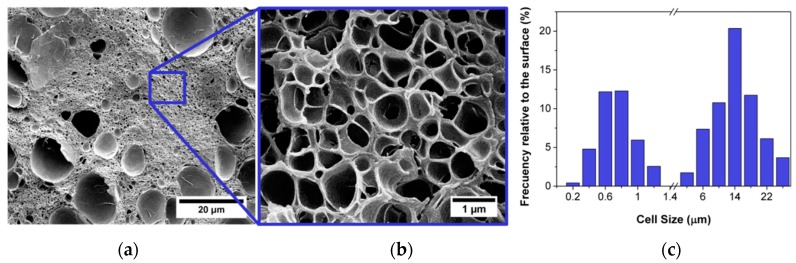
Bimodal distribution of the cell size observed for the blend of PMMA with a 0.5 wt.% of S-QAS. (**a**) General Image. (**b**) Zoom of the general image showing the nanostructure. (**c**) Cell size distribution diagram that has been calculated taking into account the different areas occupied by each population of cells (details can be found elsewhere [70]).

Results show that among the three types of sepiolites used, only that modified with a quaternary salt (S-QAS) has an effect as a nucleating agent for a particle concentration of 0.5 wt.%. Whereas the blends with 0.5 wt.% of the other sepiolites present similar cellular structures as the pure polymer, the blend with 0.5 wt.% of S-QAS shows an entirely different behaviour. To begin with, this material presents a bimodal cellular structure with micro and nanometric cells, with the nanometric population being predominant (Figure 4). Moreover, these structures can be obtained using mild processing conditions (saturation pressure of 10 MPa at 25 °C versus the 30 MPa required to obtain nanometric cells in the pure PMMA).

The reason behind the different behaviour of the sepiolite S-QAS should be related to its surface modification and its interaction with CO_2_ rather than with the dispersion of the particles. SEM (scanning electron microscopy) micrographs (carried out at the Microscopy Unit of the Scientific Park University of Valladolid by means of an Environmental Scanning Electron Microscope (ESEM), model FEI-Quanta 200FEG provided with a Schottky-Field Emission filament) of the solids and shear rheology measurements showed a similar dispersion of the sepiolites regardless their surface modification, and, for this reason, we believe that the interaction of the ammonium salt with the polymer is playing a key role in this phenomenon. 

**Figure 5:**
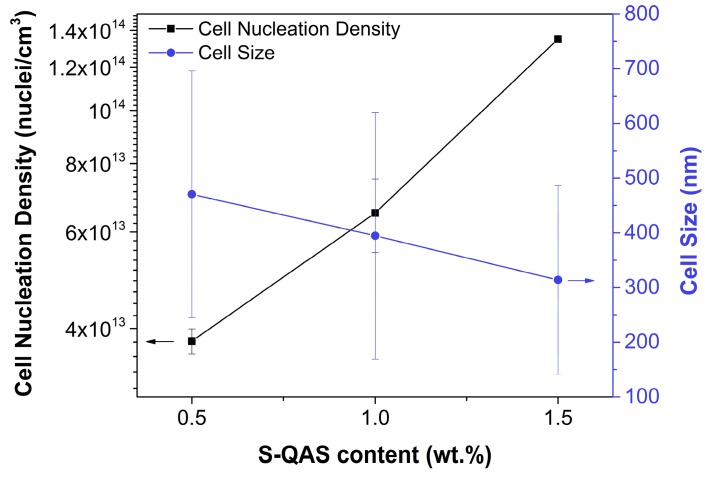
Cell nucleation density (left axis) and cell size (right axis) as a function of the sepiolite quaternary salt (S-QAS) content in the PMMA/sepiolites systems.

The effect of the addition of higher amounts of sepiolite S-QAS was also analyzed. We showed that cell nucleation density increased when particle content increased from 0.5 wt.% to 1.5 wt.% (Figure 5); that is, the higher the number of sepiolites, the larger the nucleation. Bimodal cellular structures were found for the three particle contents, with the cell size of the nanometric population decreasing for increasing particle contents. Thus, the nucleation efficiency with this sepiolite can be improved by increasing the particle content.

The alternative approach to produce nanocellular materials while taking advantage of the heterogeneous nucleation mechanism is the use of nanostructured polymers in which the size of the disperse phase is in the nanometric range [82]. In such systems, nuclei can appear in the disperse phase or in the interphase between the continuous and disperse phases. In particular, block copolymer spherical micelles gather all the qualities required to act as ideal nucleants: Nucleation is favorable in the micelles, they present uniform size and surface properties, they are easily dispersible, and the number of micelles formed is usually large [57]. To obtain nanocellular polymers with this approach, the density of the micelles of the disperse phase must be of the same order of magnitude than the cell density required. With this method, nanocellular polymers can be produced using low saturation pressures, as nucleation is controlled by the disperse phase and not by the gas dissolved into the sample [23,38,56,83].

Poly(methyl methacrylate)-poly(butyl acrylate)-poly(methyl methacrylate) (MAM) tri-block copolymer blended with PMMA produces nanostructured polymer blends with CO_2_-philic domains, suitable to produce nanocellular polymers. The effect of the copolymer content on the morphology of the blends, and thus on the cellular structure of these materials, has been widely analysed [38,56]. However, there is a lack of knowledge about the influence of the molecular weight of the copolymer in the nanostructuration of the blends, as well as in the resultant nanocellular materials.

**Figure 6:**
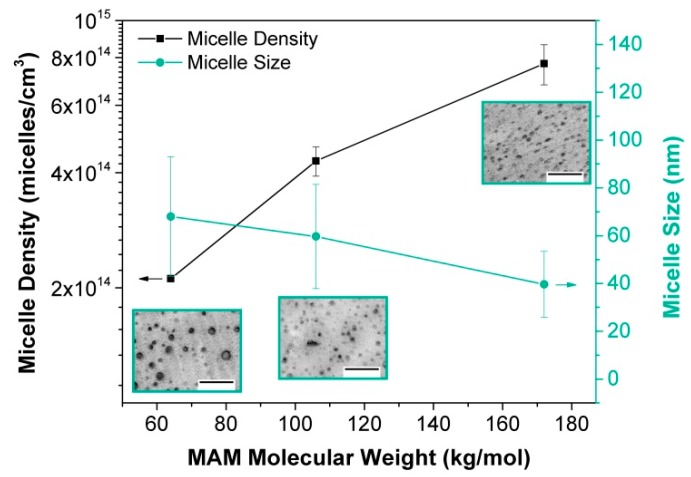
Micelle density (left axis) and micelle size (right axis) of PMMA/MAM blends as a function of the MAM molecular weight. TEM images of the blends (scale bar: 500 nm).

In our work [84], we analysed the effect of the MAM copolymer molecular weight by preparing PMMA/MAM blends with a 10 wt.% of MAM using three different grades of MAM. We observed that higher MAM molecular weights lead to higher micelle densities and smaller micelles (Figure 6).

**Figure 7:**
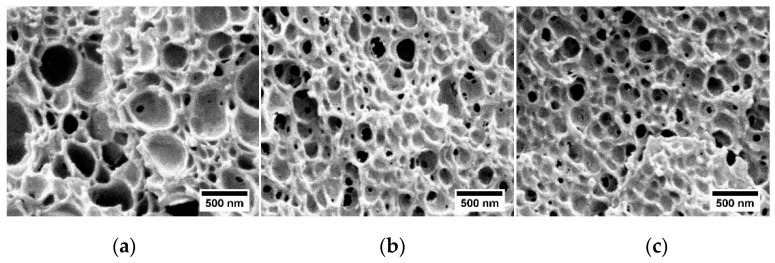
SEM micrographs of the nanocellular polymers based on PMMA/MAM blends with low (**a**), medium (**b**) and high (**c**) MAM molecular weight.

**Figure 8:**
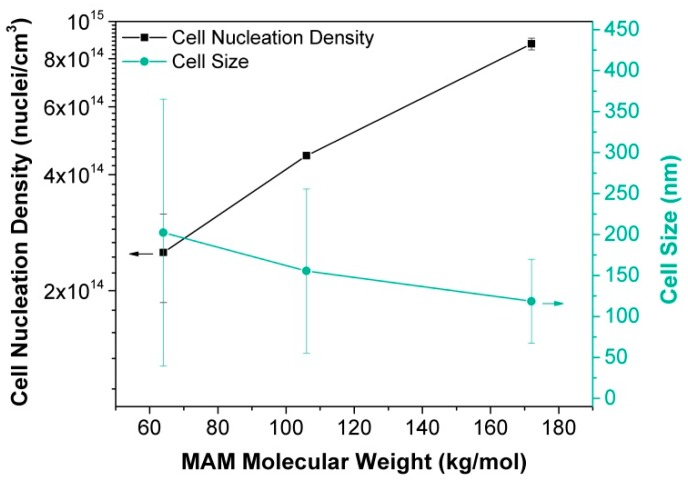
Cell nucleation density (left axis) and cell size (right axis) of PMMA/MAM blends as a function of the MAM molecular weight.

As a consequence of this nanostructuration, the cellular structures obtained with the three MAM are very different. The higher the MAM molecular weight, the higher the cell nucleation density and the smaller the cell size (Figure 7 and Figure 8). There is a good correlation between the nanostructuration in the solid blends and the cellular structure. Therefore, the MAM molecular weight can be used as a tool to control the cellular structure in these nanocellular polymers based on PMMA/MAM blends.

### 2.2. Growing and Degeneration Mechanisms

The diffusion of the nucleation gas in the gas/polymer melt to the nuclei causes cell growth. Equation (9) is the simplified equation governing the growth rate of the pores [48,53,54], where η is the viscosity of the gas/polymer mixture.

(9) $$\frac{dR}{dt}=\frac{\Delta p}{4\eta }-\frac{\gamma }{2\eta }$$

Thus, the viscosity of the polymer plays an important role, not only during nucleation but also along the growing process [48]. Taking into consideration the previous equation, the viscosity of the polymer at the processing temperatures should be low enough to allow the expansion at the initial stages of the foaming, but, on the other hand, it should be high enough to avoid coalescence mechanisms to take place. Therefore it is a parameter that should be optimized. The evolution of Equation (8) will also be affected by the diffusivity of the gas in the polymer, as it will determine the pressure gradient in every instant. Besides, both the viscosity and the surface tension depend on the gas concentration as the gas acts as a plasticizer [38,62], so they will evolve in time as the gas diffuses from the polymer to the cells and out of the material. Growing will occur as long as the temperature is higher than the effective glass transition temperature—that is, as long as the polymer is in the rubbery state. The effective glass transition temperature will increase with time as gas diffuses out the polymer.

In summation, several mechanisms take place during cell growth, all of them correlated with gas diffusion. Therefore, the diffusivity (measured in cm^2^/s), which evaluates the velocity of gas diffusion into the polymer, is an important parameter to take into account in the gas dissolution foaming of nanocellular polymers. In this sense, the fact that the cell wall thickness in the nanocellular foams are very thin could play an important role, because it could increase the speed of gas diffusion. 

During the growing of the cells, degeneration mechanisms may appear. The starting point is the opening of the cell walls that finally lead to coalescence. The opening of the cell walls could inhibit the reduction of the relative density, as was discussed in a previous work [16]. Then this opening evolves into degeneration mechanisms such as coalescence or coarsening. These effects might happen at the initial states of the foaming process; that is, nuclei can coalesce, provoking a reduction of the final cell density of the material. However, degeneration mechanisms can also appear at the end of the foaming process. For instance, in PMMA, it was found that foaming at 110 °C during 5 min led to the degeneration of the cellular structure, causing an increase in the density—probably due to the high temperature—very close to the glass transition of the polymer [16]. In systems filled with particles, their presence in the cell walls can cause cell wall ruptures when attempting to the reduce the density, as we observed in PMMA/sepiolite systems foamed at high temperatures [70]. Therefore, a fine adjustment of the foaming parameters must be performed to avoid all possible degeneration mechanisms that could lead to a reduction of the cell density and an increase of the cell size.

**Figure 9:**
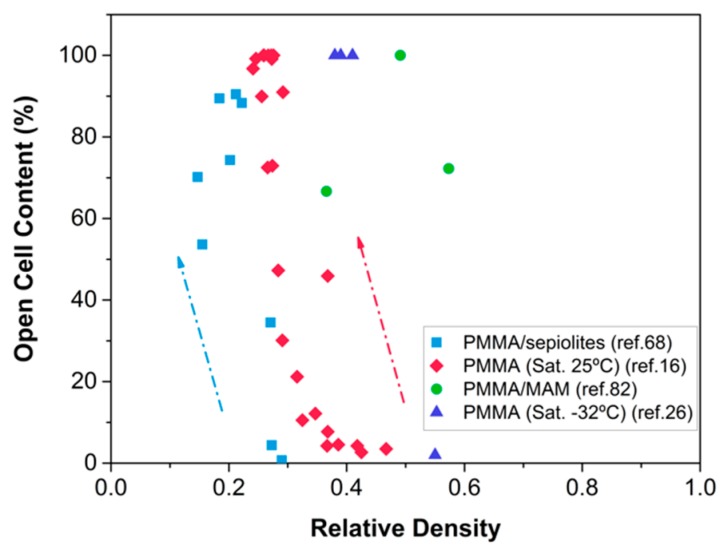
Open cell content as a function of the relative density for the different nanocellular polymers produced in our laboratory. Arrows indicate the increase of the foaming temperature.

Figure 9 shows the open cell content of some of the samples based on the different systems we have investigated so far (PMMA, PMMA filled with sepiolites, and PMMA/MAM nanostructured blends) as a function of their relative density. We observed that in the PMMA saturated at high pressure and room temperature (30 MPa, 25 °C), a reduction of the density implies an increase of the open cell content, and, once the maximum open cell content is, reached, expansion stops. Regarding the samples produced at low temperature (−32 °C), they present an open cell structure even at high relative densities, so it is plausible to assume that the opening of the cells in these systems is limiting a further expansion. In the heterogeneous systems, however, the mechanisms are intrinsically different. For instance, the PMMA/sepiolite systems show a close structure at low relative density (around 0.3), but the open cell content increases with increasing foaming temperature. However, a 100% open cell content is not achieved in these systems, because there is a microcellular population formed by closed cells. In PMMA/MAM blends, open cell structures (open cell content 60–100%) can be observed even though the densities are high. This is because the expansion mechanisms in this system are different. As a consequence of the micelle spherical shape, cell growth is restricted to the micelle-micelle distance and the minimum cell wall thickness. Once this thickness is reached, cells break and expansion stops. No further cell growth is allowed because cells are restricted to grow spherically and they cannot form polygonal shapes to fill the space.

In general terms, it can be said that less research has been conducted on this important topic of growing and degeneration mechanism in nanocellular polymers. Therefore, there are still many fundamental aspects that are unknown.

### 2.3. Skin Formation

From the moment pressure is released, gas in the surface of the material diffuses out of the sample [85,86]. As a consequence of this diffusion, there is a concentration gradient throughout the sample thickness. Near the surface the gas concentration will be very low, and, thus, the T_g,eff_ will be high. As a result, a solid skin without any pores appears near the surface [85,86]. If the foaming process is a one-step, one should expect a thin skin, as there is no time gap between the release of the pressure and the start of the expansion. However, in a two-step foaming process, in which there is a finite time between the depressurization and the foaming, the skin thickness may be significant. In fact, desorption time has been used as a parameter to control the skin thickness and the surface quality of the samples [87]. 

Some issues related to skin formation may affect the cellular structure of the nanocellular polymers. First, there is a gas concentration gradient, which could result is a nucleation gradient according to Equation (5). Therefore, desorption time should be controlled so as to avoid such an inhomogeneous structure. Besides, when foaming thin samples, desorption becomes more relevant. For instance, the foaming of films requires the use of solid constraint to avoid gas scape [68,69,88]. Finally, it is possible that a very thick solid skin can act as limiting factor of the expansion.

## 3. Limits and Future Trends

**Figure 10:**
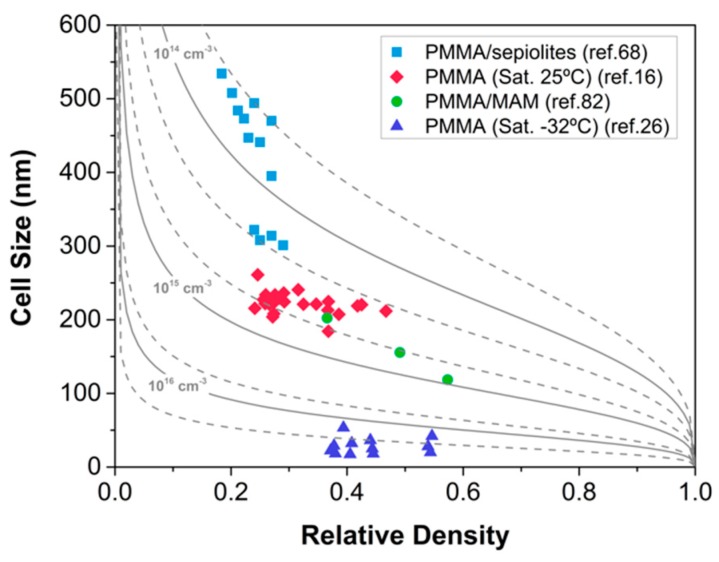
Relative density—cell size map. For the PMMA/sepiolites bimodal cellular materials [70], cell size plotted is that of the nanocellular region. Curves indicate regions of constant cell nucleation density according to Equations (1) and (2).

The results reported in this work are shown in Figure 10. The materials produced with different systems and using different strategies cover a wide range of cell sizes and relative densities. It is interesting to point out that, even if the approaches to produce the materials are different, most of them are in the same range of densities and cell sizes. For instance, the nanocellular polymers based on PMMA and produced using a saturation process at room temperature present cell sizes in the same range as the materials produced using nanostructured polymers. Slightly higher cell sizes are obtained by using the PMMA systems containing nanoparticles that seems to be less effective in nucleation than the other strategies tested. However, materials with reduced densities can be produced using this approach. Finally, the materials that present the lower cell sizes are those produced using low saturation temperatures; these materials present a completely different behavior with very small cell sizes, a very high open cell content, and higher relative densities. 

However, despite the up-to-date efforts and the different approaches followed, our results do not show materials with densities clearly below 0.2.

The limits we have found in PMMA and PMMA-based systems are in the same line as the results obtained in other laboratories. For instance, the works of Costeux et al. [48] with PMMA and PMMA copolymers reported cell sizes in the range of 100 nm with densities from 0.7 to 0.18. Guo et al. [39] obtained cell sizes of around 40 nm by reducing the saturation temperature. In the case of PMMA/MAM blends, Forest and co-workers [38] produced nanocellular samples with medium densities (around 0.4–0.5) and cell sizes from 70–300 nm. Finally, for systems with nanoparticles, the most promising results were obtained by Costeux [55], who blended PMMA with silica particles, obtaining relative densities as low as 0.15 while keeping the cell size in the nanoscale. Compared to our results reported in Figure 10, the general trend shows that there is a lack of materials in the region of very low densities and very low (under 100 nm) cell sizes that would be interesting for some applications, such as thermal insulation.

However, despite the limits that have appeared with PMMA and the fact that there is still a long way to go in order to obtain the optimum nanocellular PMMA for some applications, it is important to highlight that, in comparison with other nanocellular polymers in the bibliography, PMMA is doubtless one of the most promising systems. For instance, nanocellular PC produced by Guo et al. [31] is also an interesting system providing cellular materials with cell sizes as small as 31 nm combined with a relative density of 0.4. However smaller cell sizes or higher cell nucleation density have never been reported with this polymer matrix. Other aforementioned systems are far away from showing results comparable with nanocellular PC or nanocellular PMMA. For example, with PEI cell sizes of 30 nm have been achieved, but cell nucleation densities are not so high (around 10^14^ nuclei/cm^3^) [89]. PPSU has been proven to present also small cell sizes (21 nm), but the cell nucleation density in this material is still small in comparison with the PMMA, and its relative density is also higher (0.7) [34]. Finally, for nanocellular materials based on thermoplastic polyurethane, the smaller reported cell size is 450 nm with relative densities as high as 0.94 [32].

The challenge of reducing the density for nanocellular materials is related to all the limitations mentioned above. Different approaches to further reduce the density can be followed. On the one hand, the viscosity of the polymer influences the growth, so an attempt to reduce the density can be done by reducing the viscosity of the polymer matrix. However, the viscosity of the polymer should be high enough to avoid coalescence of the nearly formed nuclei. Therefore, it is expected that an optimum viscosity should exists. In fact, some works have shown that reducing the viscosity of the polymer matrix does lead to lower densities; however, this is done at the expense of increasing the cell size [48,84,90,91]. As such, the strategy of manipulating the viscosity of the polymer is promising and can produce interesting results in the future, but it must be carefully evaluated. 

Density is also determined by the processing parameters, such as the foaming temperature [16]. Works in literature cover a wide range of processing parameters, but there is room yet for some new research, such as the use of different post-foaming treatments in which for instance external pressure is controlled and the use of intermediate saturation temperatures (around 0 °C) combined with high pressures.

In the area of heterogeneous nucleation, inorganic nanoparticles have shown to be an up-and-coming nucleating agent for the production of materials with medium-low density [55,70]. Obtaining new nanocomposites with well-dispersed nanoparticles able to produce nanocellular polymers at mild conditions will be one of the future working lines in the development of nanocellular polymers. In the same way, the use of block copolymers is exciting, but it is still challenging to obtain low-density materials from these systems. There is a lack of understanding of the mechanisms involved in the growing of these materials, so further research is necessary to fully comprehend the possibilities of this approach. Future investigation lines in this area may include studying new, untested nanostructured systems or mixing copolymers with different viscosities.

Another challenge in the production of nanocellular materials is the size of the samples. The gas dissolution foaming process usually requires high saturation times, so thickness of the samples are limited by this parameter. Furthermore, lab pressure vessels are also limited in size; as such, the size of the initial solid samples is a boundary for the final size of the nanocellular material. In addition, foaming usually takes place in a non-constrained way (in the pressure vessel for the one-step foaming and in a thermal bath for the two-step foaming process). As a result, samples expand freely in the three dimensions, which often results in non-flat and non-homogeneously foamed samples. Future work must focus on the production of large and homogeneous samples, which is required to measure the physical properties of these materials and prove their technological interest in situations more closely related to real applications.

## 4. Conclusions

PMMA can be used as polymer matrix to produce nanocellular polymers via gas dissolution foaming using both the homogeneous and the heterogeneous nucleation strategies. In case of the homogeneous nucleation approach, we have proven that PMMA can be used to produce cell sizes of around 200 nm with a wide range of densities by tuning the foaming parameters. In addition, increasing the amount of gas dissolved of the polymer by reducing the saturation temperature allows a drastic decrease of the cell size to the range of 14–50 nm, which leads to the appearance of semi-transparent nanocellular polymers.

The use of nucleating species is the other method to obtain nanocellular structures. In particular, the use of nanometric sepiolites results in bimodal nanocellular polymers, in which the cells in the nanoscale region presents sizes ranging 300–550 nm. The increase of the sepiolite concentration induces a larger nucleation density and, thus, a smaller cell size. Regarding the use of nanostructured polymer blends, we have proven that the molecular weight of the block copolymer in PMMA/MAM blends can be used as a tool to control the cellular structure in these systems; using this makes possible to obtain cell sizes between 120 and 200 nm using the same concentration of MAM (10 wt.%).

In short, PMMA is an interesting matrix to work with for the production of nanocellular polymers. It leads to promising cellular structures for both homogeneous and heterogeneous nucleation. By controlling the type of nucleation, the nucleating agent, and the production parameters, a vast range of cell sizes, cell nucleation densities, and relative densities have been covered. However, it is mandatory to study more in-depth these systems for the production of materials combining very low-density with tiny cell sizes and to develop strategies and methodologies that could produce larger parts of these materials.
